# Author Correction: Testing for context-dependent effects of prenatal thyroid hormones on offspring survival and physiology: an experimental temperature manipulation

**DOI:** 10.1038/s41598-021-85657-w

**Published:** 2021-03-17

**Authors:** Bin-Yan Hsu, Tom Sarraude, Nina Cossin-Sevrin, Mélanie Crombecque, Antoine Stier, Suvi Ruuskanen

**Affiliations:** 1 Section of Ecology, Department of Biology, University of Turku, Turku, Finland; 2 GELIFES, University of Groningen, Groningen, The Netherlands; 3 Institute of Biodiversity, Animal Health, and Comparative Medicine, University of Glasgow, Glasgow, UK

Correction to: *Scientific Reports* https://doi.org/10.1038/s41598-020-71511-y, published online 03 September 2020

This Article contains an error in Figure 2: the y axis labels for panels (b) and (c) mistakenly use the unit “pg/ml”, when the correct unit is “ng/ml”. As a result, the Figure legend,

“Effects of prenatal hormone manipulation (TH = experimentally elevated yolk thyroid hormone treatment, CO = control) and postnatal temperature manipulation (non-heated vs. heated nests) on offspring phenotype and physiology. (a) Nestling body mass growth pattern (g, average ± SE); (b) plasma triiodothyronine (T3) concentration (pg/ml, marginal means ± SE), (c) plasma thyroxine (T4) concentration (pg/ml, marginal means ± SE), (d) mitochondrial density in blood cells (ln-transformed, marginal means ± SE), (e) blood total glutathione concentration (tGSH, nmol/mg protein, ln-transformed means ± SE) and (f) lipid peroxidation (MDA concentration, µmol/mg protein, ln-transformed means ± SE). Heated nests were on average 2.75 °C warmer than non-heated ones. See text and ESM for details on statistics and sample sizes.”

should read:

“Effects of prenatal hormone manipulation (TH = experimentally elevated yolk thyroid hormone treatment, CO = control) and postnatal temperature manipulation (non-heated vs. heated nests) on offspring phenotype and physiology. (a) Nestling body mass growth pattern (g, average ± SE); (b) plasma triiodothyronine (T3) concentration (ng/ml, marginal means ± SE), (c) plasma thyroxine (T4) concentration (ng/ml, marginal means ± SE), (d) mitochondrial density in blood cells (ln-transformed, marginal means ± SE), (e) blood total glutathione concentration (tGSH, nmol/mg protein, ln-transformed means ± SE) and (f) lipid peroxidation (MDA concentration, µmol/mg protein, ln-transformed means ± SE). Heated nests were on average 2.75 °C warmer than non-heated ones. See text and ESM for details on statistics and sample sizes.”

The correct Figure 2b-c and the correct Figure legend appear below as Figure 1.

**Figure 1:**
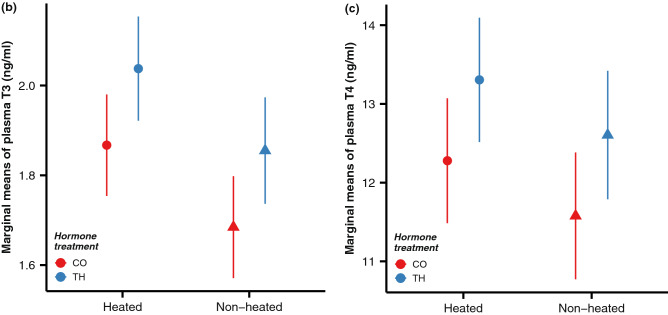
A correct version of the original Figure 2b-c. Effects of prenatal hormone manipulation (TH = experimentally elevated yolk thyroid hormone treatment, CO = control) and postnatal temperature manipulation (non-heated vs. heated nests) on offspring phenotype and physiology. (**a**) Nestling body mass growth pattern (g, average ± SE); (**b**) plasma triiodothyronine (T3) concentration (ng/ml, marginal means ± SE), (**c**) plasma thyroxine (T4) concentration (ng/ml, marginal means ± SE), (**d**) mitochondrial density in blood cells (ln-transformed, marginal means ± SE), (**e**) blood total glutathione concentration (tGSH, nmol/mg protein, ln-transformed means ± SE) and (**f**) lipid peroxidation (MDA concentration, µmol/mg protein, ln-transformed means ± SE). Heated nests were on average 2.75 °C warmer than non-heated ones. See text and ESM for details on statistics and sample sizes.

