# Supplementary material for: Testing for context-dependent effects of prenatal thyroid hormones on offspring survival and physiology: an experimental temperature manipulation
Source: Sci Rep. 2020 Sep 3;10:14563. doi: 10.1038/s41598-020-71511-y (PMC7471313; doi:10.1038/s41598-020-71511-y)
Supplement: Supplementary file 1 — Supplementary information [file 41598_2020_71511_MOESM1_ESM.docx]

Supplementary material to Hsu et al. **Context-dependent effects of prenatal thyroid hormones on offspring growth and physiology: an experimental temperature manipulation**

Bin-Yan Hsu*^1^, Tom Sarraude^1,2^, Nina Cossin-Sevrin^1^, Melanie Crombecque^1^, Antoine Stier^§1,3^ & Suvi Ruuskanen^§1^

^1^ Section of Ecology, Department of Biology, University of Turku, Finland

^2^ GELIFES, University of Groningen, The Netherlands

^3^ Institute of Biodiversity, Animal Health, and Comparative Medicine, University of Glasgow, UK

**Mitochondrial density and molecular sexing analyses by qPCR**

We extracted DNA from red blood cell pellet using a standard salt extraction alcohol precipitation method (Aljanabi and Martinez 1997). Extracted DNA was diluted in elution buffer BE for DNA preservation. DNA concentration and quality (260/280 and 260/230 ratios) were checked with a ND-1000-Spectrophotometer (NanoDrop Technologies, Wilmington, USA), and DNA integrity was verified in 48 samples chosen randomly using gel electrophoresis (50 ng of DNA, 0.8 % agarose gel at 100 mV for 60 min) and DNA staining with Midori Green. Each sample was then diluted to a concentration of 1.2 ng/µl for subsequent qPCR analysis.

Relative mitochondrial DNA copy number (mtDNAcn, an index of mitochondrial density) was quantified using a real-time quantitative PCR (qPCR) assays previously used and validated in this species (Stier et al. 2019). This technique estimates relative mtDNAcn as the ratio between one mitochondrial gene and one single copy nuclear gene. Here, we used RAG1 as a SCG (verified as single copy using a BLAST analysis on the collared flycatcher *Ficedula albicollis* genome) and cytochrome oxidase subunit 2 (COI2) as a mitochondrial gene (verified as non-duplicated in the nuclear genome using a BLAST analysis on the collared flycatcher *Ficedula albicollis* genome). Forward and reverse RAG1 primers were 5'-GCAGATGAACTGGAGGCTATAA-3' and 5'-CAGCTGAGAAACGTGTTGATTC-3', respectively. Forward and reverse COI2 primers were 5’-GGAGACGACCAAGTCTACAATG-3’; 5’-TTTCCGAACCCTCCGATTATG-3’, respectively. For the qPCR assays, the reactions were performed on a 384-QuantStudio™ 12K Flex Real-Time PCR System (Thermo Fisher), in a total volume of 12µL including 6ng of DNA, primers at a final concentration of 200nM and 6μL of Absolute Blue qPCR Mix SYBR Green low ROX (Thermo Scientific). RAG1 and COI2 reactions were performed in triplicates on the same plates (6 plates in total); the qPCR conditions were: 15 min at 95°C, followed by 35 cycles of 15 s at 95°C, 30 s at 60°C and 30s at 72°C. A DNA sample being a pool of DNA from 10 individuals was used as a reference sample and was included in triplicate on every plate. The efficiency of each amplicon was estimated from a standard curve of the reference sample ranging from 1.5 to 24ng. The mean reaction efficiencies were 101.7 ± 2.8% for RAG1, 95.5 ± 1.8% for COI2. The relative mtDNAcn of each sample was calculated as (1+*Ef*_COI2_)^ΔCq COI2^/(1+*Ef*_RAG1_)^ΔCqRAG1^; *Ef* being the amplicon efficiency, and ΔCq the difference in Cq-values between the reference sample and the focal sample. Intra-plate technical repeatability based on triplicates was 0.93 (95% C.I. [0.91-0.94]; N = 262). Inter-plate technical repeatability based on a few samples repeated on different plates was 0.96 (95% C.I. [0.86-0.99]; N = 12). The use of mtDNAcn as an index of mitochondrial density has been criticized in human (Larsen et al. 2012), but we have previously shown good correlations between mtDNAcn and mitochondrial respiration rates in pied flycatcher adult females (Stier et al. 2019).

Chicks were molecularly sexed using a qPCR approach adapted from Ellegren et al. 1997 and Chang et al. 2008. Forward and reverse sexing primers were 5′- CACTACAGGGAAAACTGTAC-3′ (2987F) and 5′- CCCCTTCAGGTTCTTTAAAA -3′ (3112R), respectively. qPCR reactions were performed in a total volume of 12µL including 6ng of DNA, primers at a final concentration of 800nM and 6μL of SensiFAST^TM^ SYBR® Lo-ROX Kit (Bioline). qPCR conditions were: 3 min at 95°C, followed by 40 cycles of 45 s at 95°C, 60 s at 52°C and 60s at 72°C, then followed by a melting curve analysis (95°C 60s, 45°C 50s, increase to 95°C at 0.1°C/s, 95°C 30s). Samples were run in duplicates in a single plate and 6 adults of known sex were included as positive controls. Sex was determined by looking at the dissociation curve, with two peaks indicating the presence of a Z and W chromosome (female), and one peak indicating the presence of only the Z chromosomes (male).

**Interaction between elevated yolk THs and actual nest-box temperature**

On average, our heating treatment raised the nest-box temperature by 2.75 °C (see main text and Fig. S1). Nevertheless, substantial variation in temperature exist among nest-boxes (ranges: average temperature 13.94 – 23.04 °C, minimum temperature 11.79 – 21.43 °C, maximum temperature 16.20 – 25.90 °C). Therefore, the true temperature-dependent effects of yolk THs, if any, might only manifest if we looked at the interaction with the actual nest temperature instead of the two-level heating treatment. To address this, we repeated all of our models (Table S1-S7) with the heating treatment being replaced by either the average, minimum, or maximum nest-box temperature across the period of heating treatment (day 2 to day 8 after hatching). The minimum nest-box temperature positively predicted nestling fledging success, but TH-nestings did not show increased fledging success compared to CO-nestlings (see main text and Fig. 1). In contrast, the average and maximum nest-box temperatures did not show any clear effects or interactions with yolk TH treatment on fledging success. For the rest of the models, in agreement with the models that only considered heating treatment, we generally found no clear effects of THs and temperature on offspring traits. There were, however, two statistically significant interactions (i.e. p < 0.05): One suggested that at day 13, TH-nestlings were lighter (adjusted slope = -0.119 ± 0.100 g/°C) whereas CO-nestlings got heavier (adjusted slope = 0.075 ± 0.094 g/°C) as the average nest temperature during day 2 to day 8 increased (p=0.043, Fig. S3 left panel). The other suggested that at day 13, TH-nestlings had higher blood MDA concentrations (adjusted slope = 0.030 ± 0.017 on ln scale) whereas the blood MDA concentrations in CO-nestlings barely changed (adjusted slope = -0.005 ± 0.016 on ln scale) as the maximum nest-box temperature increased (p = 0.026, Fig. S3 right panel). Nevertheless, we have strong reservation on the authenticity and biological relevance of these potential effects: As shown in Fig. S3, we plotted the residuals from the models that excluded the involving factors to visualize the effects. While the adjusted slopes across temperature were opposite between TH- and CO-nestlings, the scattering of the data points hardly demonstrate the difference. We therefore still conclude that we did not find convincing evidence for temperature-dependent effects of yolk THs.

**Table S1**. Generalized linear mixed models (GLMM) on the effects of prenatal thyroid hormone elevation (TH/Control) and postnatal temperature elevation (heated/non-heated) on nestling fledging success. Nestlings that died before sufficient exposure to heating treatment (i.e. before d4, n=11) were excluded from the analysis. When using nest-box temperature as the predictor (see next page), the sample size was smaller due to thermologger malfunctions in four boxes. The models were fit by maximum likelihood approach using Laplace approximation with binomial distribution and logit link function. Brood size, date, initial mass (d2) were included as covariates, and nest of origin and rearing as random intercepts. Main effects were reported from a model with no interactions.

| (A) Fledging success, using heating treatment as the predictor (n=236) | | | | |
| --- | --- | --- | --- | --- |
| Random effects: |  | **Variance** | **Std. Dev.** |  |
| Nest of origin (n=56) | Intercept | 0.731 | 0.855 |  |
| Nest of rearing (n=56) | Intercept | 17.879 | 4.228 |  |
| Fixed factors | **Estimate** | **SE** | **z** | **p** |
| Intercept | 3.624 | 3.705 | 0.978 |  |
| Hormone (TH) | 0.392 | 0.765 | 0.513 | 0.608 |
| Heating (Non-heated) | 1.534 | 1.662 | 0.923 | 0.356 |
| Brood size | -1.624 | 0.764 | -2.127 | **0.033** |
| Body mass | 2.126 | 0.680 | 3.127 | **0.002** |
| Date | -3.491 | 1.271 | -2.747 | **0.006** |
| Hormone × Heating | 0.196 | 1.259 | 0.156 | 0.876 |
| N = 76 CO-non-heated, 50 CO-heated, 58 TH-non-heated, 52 TH-heated. | | | | |

**Table S1 (cont.)**

| (B) Fledging success, using average nest temperature as the predictor (n=223) | | | | |
| --- | --- | --- | --- | --- |
| Random effects: |  | **Variance** | **Std. Dev.** |  |
| Nest of origin (n=52) | Intercept | 0.743 | 0.862 |  |
| Nest of rearing (n=52) | Intercept | 18.809 | 4.337 |  |
| Fixed factors | **Estimate** | **SE** | **z** | **p** |
| Intercept | -8.570 | 10.080 | -0.850 |  |
| Hormone (TH) | 0.324 | 0.768 | 0.422 | 0.673 |
| Average nest temperature | 0.681 | 0.485 | 1.403 | 0.161 |
| Brood size | -1.653 | 0.771 | -2.145 | **0.032** |
| Body mass | 2.152 | 0.715 | 3.011 | **0.003** |
| Date | -3.618 | 1.210 | -2.990 | **0.003** |
| Hormone × Temperature | -0.189 | 0.513 | -0.368 | 0.713 |
| (C) Fledging success, using minimum nest temperature as the predictor (n=223) | | | | |
| Random effects: |  | **Variance** | **Std. Dev.** |  |
| Nest of origin (n=52) | Intercept | 0.770 | 0.878 |  |
| Nest of rearing (n=52) | Intercept | 13.348 | 3.654 |  |
| Fixed factors | **Estimate** | **SE** | **z** | **p** |
| Intercept | -15.936 | 8.433 | -1.890 |  |
| Hormone (TH) | 0.237 | 0.756 | 0.314 | 0.754 |
| Minimum nest temperature | 1.181 | 0.485 | 2.438 | **0.015** |
| Brood size | -1.383 | 0.690 | -2.006 | **0.045** |
| Body mass | 2.135 | 0.694 | 3.077 | **0.002** |
| Date | -3.732 | 1.145 | -3.260 | **0.001** |
| Hormone × Temperature | 0.208 | 0.456 | 0.455 | 0.649 |
| (D) Fledging success, using maximum nest temperature as the predictor (n=223) | | | | |
| Random effects: |  | **Variance** | **Std. Dev.** |  |
| Nest of origin (n=52) | Intercept | 0.500 | 0.707 |  |
| Nest of rearing (n=52) | Intercept | 16.996 | 4.123 |  |
| Fixed factors | **Estimate** | **SE** | **z** | **p** |
| Intercept | 7.174 | 10.106 | 0.710 |  |
| Hormone (TH) | 0.285 | 0.729 | 0.391 | 0.696 |
| Maximum nest temperature | -0.074 | 0.398 | -0.185 | 0.853 |
| Brood size | -1.814 | 0.780 | -2.326 | **0.020** |
| Body mass | 2.006 | 0.664 | 3.021 | **0.003** |
| Date | -3.426 | 1.256 | -2.727 | **0.006** |
| Hormone × Temperature | -0.054 | 0.385 | -0.139 | 0.889 |

**Table S2**. General linear mixed model (GLMM) on the effects of prenatal thyroid hormone elevation (TH/Control) and postnatal temperature elevation (heated/non-heated) on body mass at (a) day 2, prior to heating treatment; (b) d8, after heating treatment and (c) d13 (close to fledging) after hatching. Brood size, date, initial mass (d2) and cross-fostering status (cross-fostered or not) have been included as covariates, and nest of origin and rearing as random intercepts when applicable. Sex was determined at d13. Main effects were reported from a model with no interactions. The significance tests were conducted using Kenward-Roger approximation on the degrees of freedom.

| (a) Day-2 body mass (n=248) | | | | | | |
| --- | --- | --- | --- | --- | --- | --- |
| Random effects: |  | **Variance** | **Std. Dev.** |  |  |  |
| Nest of origin (n=58) | Intercept | 0.326 | 0.571 |  |  |  |
| Residual |  | 0.339 | 0.583 |  |  |  |
| Fixed effects: | **Estimate** | **Std. Error** | **t** | **df** | **F** | **p** |
| Intercept | -138.700 | 185.800 | -0.747 |  |  |  |
| Hormone (TH) | -0.193 | 0.170 | -1.140 | 1, 54.23 | 1.299 | 0.259 |
| Date | 0.008 | 0.011 | 0.765 | 1, 55.74 | 0.584 | 0.448 |
| (b) Day-8 body mass (n=209) | | | | | | |
| Random effects: |  | **Variance** | **Std. Dev.** |  |  |  |
| Nest of origin (n=54) | Intercept | <0.001 | <0.001 |  |  |  |
| Nest of rearing (n=52) | Intercept | 0.839 | 0.916 |  |  |  |
| Residual |  | 0.605 | 0.778 |  |  |  |
| Fixed factors | **Estimate** | **SE** | **t** | **df** | **F** | **p** |
| Intercept | 1518.487 | 329.132 | 4.614 |  |  |  |
| Hormone (TH) | -0.075 | 0.131 | -0.571 | 1, 21.90 | 0.317 | 0.579 |
| Heating (Non-heated) | -0.014 | 0.295 | -0.046 | 1, 46.80 | 0.002 | 0.963 |
| Brood size | -0.265 | 0.124 | -2.135 | 1, 50.36 | 4.543 | **0.038** |
| Cross-foster (yes) | 0.218 | 0.121 | 1.805 | 1, 152.50 | 3.224 | 0.075 |
| Day-2 body mass | 1.109 | 0.092 | 12.113 | 1, 112.55 | 137.660 | **<0.001** |
| Date | -0.085 | 0.019 | -4.587 | 1, 21.01 | 47.690 | **<0.001** |
| Hormone × Heating | 0.311 | 0.257 | 1.210 | 1, 172.68 | 1.432 | 0.233 |

| (c) Day-13 body mass (n=183) | | | | | | |
| --- | --- | --- | --- | --- | --- | --- |
| Random effects: |  | **Variance** | **Std. Dev.** |  |  |  |
| Nest of origin (n=53) | Intercept | 0.316 | 0.562 |  |  |  |
| Nest of rearing (n=48) | Intercept | 0.766 | 0.875 |  |  |  |
| Residual |  | 0.610 | 0.781 |  |  |  |
| Fixed factors | **Estimate** | **SE** | **t** | **df** | **F** | **p** |
| Intercept | 1813.456 | 370.056 | 4.900 |  |  |  |
| Hormone (TH) | 0.173 | 0.227 | 0.763 | 1, 32.30 | 0.565 | 0.456 |
| Heating (Non-heated) | 0.041 | 0.307 | 0.133 | 1, 37.18 | 0.018 | 0.896 |
| Brood size | -0.502 | 0.133 | -3.785 | 1, 43.08 | 14.163 | **<0.001** |
| Cross-foster (yes) | -0.089 | 0.140 | -0.636 | 1, 133.01 | 0.399 | 0.529 |
| Day-2 body mass | 0.397 | 0.122 | 3.265 | 1, 149.50 | 10.231 | **0.002** |
| Date | -0.102 | 0.021 | -4.863 | 1, 50.97 | 23.582 | **<0.001** |
| Sex (male) | 0.274 | 0.142 | 1.933 | 1, 142.73 | 3.652 | 0.058 |
| Hormone × Heating | 0.442 | 0.304 | 1.457 | 1, 150.27 | 2.068 | 0.153 |
| Hormone × Sex | -0.104 | 0.281 | -0.370 | 1, 137.70 | 0.134 | 0.715 |
| Heating × Sex | 0.357 | 0.287 | 1.246 | 1, 144.58 | 1.515 | 0.220 |
| Sample sizes: Day 2: n = 132 CO, 116 TH;  Day 8: n = 60 CO-non-heated, 47 CO-heated, 53 TH-non-heated, 49 TH-heated  Day 13: n = 55 CO-non-heated, 42 CO-heated, 45 TH-non-heated, 41 TH-heated | | | | | | |

Table S2. (cont.)

**Table S3**. General linear mixed model (GLMM) on the effects of prenatal thyroid hormone elevation (TH/Control) and postnatal temperature elevation (heated/non-heated) on tarsus length at (a) d8, after heating treatment and (b) at close to fledging (d13) after hatching. Brood size, date, initial size, cross-fostering status (cross-fostered or not) and measurer have been included as covariates, and nests of origin and rearing as random intercepts. Sex was determined at d13. Main effects were reported from a model with no interactions. The significance tests were conducted using Kenward-Roger approximation on the degrees of freedom.

| (a) Day-8 tarsus length (n=209) | | | | | | |
| --- | --- | --- | --- | --- | --- | --- |
| Random effects: |  | **Variance** | **Std. Dev.** |  |  |  |
| Nest of origin (n=54) | Intercept | <0.001 | <0.001 |  |  |  |
| Nest of rearing (n=52) | Intercept | 0.503 | 0.709 |  |  |  |
| Residual |  | 0.382 | 0.618 |  |  |  |
| Fixed factors | **Estimate** | **SE** | **t** | **df** | **F** | **p** |
| Intercept | 895.106 | 257.290 | 3.479 |  |  |  |
| Hormone (TH) | -0.067 | 0.104 | -0.642 | 1, 21.95 | 0.400 | 0.534 |
| Heating (Non-heated) | 0.001 | 0.230 | 0.006 | 1, 44.83 | <0.001 | 0.996 |
| Brood size | -0.084 | 0.097 | -0.861 | 1, 48.33 | 0.739 | 0.394 |
| Cross-foster (yes) | 0.105 | 0.096 | 1.092 | 1, 152.38 | 1.180 | 0.279 |
| Day-2 body mass | 0.855 | 0.073 | 11.746 | 1, 111.52 | 129.380 | **<0.001** |
| Date | -0.050 | 0.015 | -3.419 | 1, 45.77 | 11.662 | **0.001** |
| Measurer (LB) | 0.052 | 0.371 | 0.141 | 1, 70.94 | 1.238 | 0.296 |
| Measurer (TR) | 0.376 | 0.389 | 0.966 |  |  |  |
| Hormone × Heating | 0.260 | 0.203 | 1.277 | 1, 170.63 | 1.596 | 0.208 |
| (b) Day-13 tarsus length (n=183) | | | | | | |
| Random effects: |  | **Variance** | **Std. Dev.** |  |  |  |
| Nest of origin (n=53) | Intercept | 0.053 | 0.231 |  |  |  |
| Nest of rearing (n=48) | Intercept | 0.119 | 0.345 |  |  |  |
| Residual |  | 0.152 | 0.390 |  |  |  |
| Fixed factors | **Estimate** | **SE** | **t** | **df** | **F** | **p** |
| Intercept | 587.604 | 154.375 | 3.806 |  |  |  |
| Hormone (TH) | -0.043 | 0.101 | -0.428 | 1, 32.66 | 0.177 | 0.677 |
| Heating (Non-heated) | 0.003 | 0.131 | 0.024 | 1, 35.90 | 0.001 | 0.981 |
| Brood size | -0.073 | 0.057 | -1.290 | 1, 42.41 | 1.639 | 0.207 |
| Cross-foster (yes) | -0.069 | 0.069 | -1.008 | 1, 138.44 | 0.998 | 0.320 |
| Day-2 body mass | 0.219 | 0.058 | 3.739 | 1, 148.17 | 13.324 | **<0.001** |
| Date | -0.032 | 0.009 | -3. 687 | 1, 47.46 | 13.532 | **<0.001** |
| Sex (male) | -0.019 | 0.070 | -0.279 | 1, 146.80 | 0.076 | 0.784 |
| Measurer (LB) | 0.759 | 0.445 | 1.705 | 2, 51.67 | 1.489 | 0.235 |
| Measurer (TR) | 0.784 | 0.451 | 1.741 |  |  |  |
| Hormone × Heating | 0.012 | 0.150 | 0.078 | 1, 157.73 | 0.006 | 0.939 |
| Hormone × Sex | -0.044 | 0.138 | -0.320 | 1, 143.34 | 0.100 | 0.753 |
| Heating × Sex | 0.127 | 0.140 | 0.909 | 1, 150.95 | 0.803 | 0.372 |
| Sample sizes: Day 8: n = 60 CO-non-heated, 47 CO-heated, 53 TH-non-heated, 49 TH-heated  Day 13: n = 55 CO-non-heated, 42 CO-heated, 45 TH-non-heated, 41 TH-heated | | | | | | |

**Table S4**. General linear mixed model (GLMM) on the effects of prenatal thyroid hormone elevation (TH/Control) and postnatal temperature elevation (heated/non-heated) on (a) plasma triiodothyronine (T3) and (b) thyroxine (T4) concentrations (pg/ml) in day-13 nestlings. Body mass, cross-fostering status (cross-fostered or not), nestling sex, and hormone extraction batch have been included as covariates, and nests of origin and rearing as random intercepts. Main effects were reported from a model with no interactions. The significance tests were conducted using Kenward-Roger approximation on the degrees of freedom.

| (a) Plasma T3 concentration (n=74) | | | | | | |
| --- | --- | --- | --- | --- | --- | --- |
| Random effects: |  | **Variance** | **Std. Dev.** |  |  |  |
| Nest of origin (n=43) | Intercept | 0.031 | 0.176 |  |  |  |
| Nest of rearing (n=37) | Intercept | <0.001 | <0.001 |  |  |  |
| Residual |  | 0.032 | 0.179 |  |  |  |
| Fixed factors | **Estimate** | **SE** | **t** | **df** | **F** | **p** |
| Intercept | -0.924 | 0.493 | -1.877 |  |  |  |
| Hormone (TH) | 0.171 | 0.134 | 1.275 | 1, 28.28 | 1.553 | 0.223 |
| Heating (Non-heated) | 0.183 | 0.126 | 1.443 | 1, 27.52 | 1.992 | 0.169 |
| Body mass | 0.196 | 0.035 | 5.554 | 1, 36.48 | 26.892 | **<0.001** |
| Sex (male) | 0.074 | 0.128 | 0.579 | 1, 63.18 | 0.299 | 0.587 |
| Cross-foster (yes) | -0.134 | 0.122 | -1.091 | 1, 32.44 | 1.146 | 0.292 |
| Batch (B) | -0.048 | 0.169 | -0.287 | 2, 24.03 | 0.079 | 0.924 |
| Batch (C) | -0.062 | 0.155 | -0.397 |  |  |  |
| Hormone × Heating | -0.335 | 0.237 | -1.415 | 1, 37.28 | 1.896 | 0.177 |
| Hormone × Sex | -0.318 | 0.253 | -1.259 | 1, 64.04 | 1.443 | 0.234 |
| (b) Plasma T4 concentration (n=74) | | | | | | |
| Random effects: |  | **Variance** | **Std. Dev.** |  |  |  |
| Nest of origin (n=43) | Intercept | 5.894 | 2.428 |  |  |  |
| Nest of rearing (n=37) | Intercept | <0.001 | 0.002 |  |  |  |
| Residual |  | 6.772 | 2.602 |  |  |  |
| Fixed factors | **Estimate** | **SE** | **t** | **df** | **F** | **p** |
| Intercept | 3.569 | 3.083 | 1.158 |  |  |  |
| Hormone (TH) | 1.027 | 1.001 | 1.026 | 1, 34.47 | 1.032 | 0.317 |
| Heating (Non-heated) | 0.701 | 0.722 | 0.970 | 1, 22.37 | 0.895 | 0.354 |
| Body mass | 0.563 | 0.219 | 2.573 | 1, 33.16 | 5.648 | **0.023** |
| Sex (male) | 0.203 | 0.777 | 0.261 | 1, 53.19 | 0.060 | 0.808 |
| Cross-foster (yes) | -0.669 | 0.684 | -0.979 | 1, 26.25 | 0.917 | 0.347 |
| Batch (B) | 1.438 | 1.220 | 1.178 | 2, 33.54 | 0.731 | 0.489 |
| Batch (C) | 0.278 | 1.177 | 0.236 |  |  |  |
| Hormone × Heating | -0.242 | 1.389 | -0.174 | 1, 32.08 | 0.028 | 0.867 |
| Hormone × Sex | -1.222 | 1.583 | -0.772 | 1, 60.57 | 0.537 | 0.467 |
| Heating × Sex | -0.291 | 1.531 | -0.190 | 1, 57.16 | 0.032 | 0.858 |
| Sample sizes: n = 21 CO-non-heated, 20 CO-heated, 17 TH-non-heated, 16 TH-heated | | | | | | |

**Table S5**. General linear mixed model (GLMM) on the effects of prenatal thyroid hormone elevation (TH/Control) and postnatal temperature elevation (heated/non-heated) on mitochondria density, ie. mitochondria DNA copy number, in day-13 nestlings. Mitochondria DNA copy numbers were ln-transformed to ensure a normal residual distribution. Body mass, nestling sex and cross-fostering status (cross-fostered or not) have been included as covariates, and nests of origin and rearing as random intercepts. The plate ID of qPCR was not included as a random intercept because the variance across qPCR plates was very small and led to singular fit (the intra- and inter-plate repeatabilities were both very high, see ESM). Main effects were reported from a model with no interactions. The significance tests were conducted using Kenward-Roger approximation on the degrees of freedom.

| Mitochondria DNA copy number (n=183) | | | | | | |
| --- | --- | --- | --- | --- | --- | --- |
| Random effects: |  | **Variance** | **Std. Dev.** |  |  |  |
| Nest of origin (n=53) | Intercept | 0.013 | 0.114 |  |  |  |
| Nest of rearing (n=48) | Intercept | 0.077 | 0.277 |  |  |  |
| Residual |  | 0.055 | 0.234 |  |  |  |
| Fixed factors | **Estimate** | **SE** | **t** | **df** | **F** | **p** |
| Intercept | 1.563 | 0.288 | 5.432 |  |  |  |
| Hormone (TH) | 0.049 | 0.056 | 0.879 | 1, 31.01 | 0.739 | 0.397 |
| Heating (Non-heated) | 0.005 | 0.090 | 0.053 | 1, 41.52 | 0.003 | 0.958 |
| Body mass | 0.005 | 0.020 | 0.243 | 1, 167.41 | 0.056 | 0.813 |
| Sex (male) | 0.023 | 0.042 | 0.539 | 1, 149.95 | 0.283 | 0.596 |
| Cross-foster (yes) | -0.035 | 0.041 | -0.858 | 1, 135.85 | 0.724 | 0.396 |
| Hormone × Heating | -0.069 | 0.091 | -0.755 | 1, 155.76 | 0.554 | 0.458 |
| Hormone × Sex | 0.064 | 0.081 | 0.781 | 1, 138.81 | 0.598 | 0.441 |
| Heating × Sex | 0.059 | 0.084 | 0.702 | 1, 147.56 | 0.479 | 0.490 |
| Sample sizes: n = 55 CO-non-heated, 44 CO-heated, 45 TH-non-heated, 39 TH-heated | | | | | | |

**Table S6**. General linear mixed model (GLMM) on the effects of prenatal thyroid hormone elevation (TH/Control) and postnatal temperature elevation (heated/non-heated) on blood total gluthathione concentration (tGSH) at (a) d8 and (b) d13 after hatching. Body mass and cross-fostering status (cross-fostered or not) have been included as covariates, and nests of origin and rearing as random intercepts. Sex was determined at d13. Main effects were reported from a model with no interactions. The significance tests were conducted using Kenward-Roger approximation on the degrees of freedom.

| (a) Day-8 tGSH (n=173) | | | | | | |
| --- | --- | --- | --- | --- | --- | --- |
| Random effects: |  | **Variance** | **Std. Dev.** |  |  |  |
| Nest of origin (n=45) | Intercept | 0.004 | 0.067 |  |  |  |
| Nest of rearing (n=44) | Intercept | 0.008 | 0.089 |  |  |  |
| tGSH assay (n=4) | Intercept | 0.009 | 0.093 |  |  |  |
| Residual |  | 0.108 | 0.328 |  |  |  |
| Fixed factors | **Estimate** | **SE** | **t** | **df** | **F** | **p** |
| Intercept | 0.159 | 0.244 | 0.649 |  |  |  |
| Hormone (TH) | -0.073 | 0.056 | -1.293 | 1, 30.90 | 1.606 | 0.215 |
| Heating (Non-heated) | -0.038 | 0.058 | -0.651 | 1, 29.87 | 0.415 | 0.524 |
| Body mass | -0.021 | 0.019 | -1.105 | 1, 110.11 | 1.137 | 0.289 |
| Cross-foster (yes) | 0.045 | 0.051 | 0.874 | 1, 134.61 | 0.751 | 0.388 |
| Hormone × Heating | -0.089 | 0.106 | -0.838 | 1, 155.85 | 0.673 | 0.413 |
| (b) Day-13 tGSH (n=149) | | | | | | |
| Random effects: |  | **Variance** | **Std. Dev.** |  |  |  |
| Nest of origin (n=45) | Intercept | <0.001 | <0.001 |  |  |  |
| Nest of rearing (n=39) | Intercept | 0.020 | 0.141 |  |  |  |
| tGSH assay (n=4) | Intercept | 0.023 | 0.153 |  |  |  |
| Residual |  | 0.032 | 0.179 |  |  |  |
| Fixed factors | **Estimate** | **SE** | **t** | **df** | **F** | **p** |
| Intercept | -0.377 | 0.207 | -1.819 |  |  |  |
| Hormone (TH) | 0.014 | 0.033 | 0.411 | 1, 21.12 | 0.161 | 0.692 |
| Heating (Non-heated) | -0.007 | 0.055 | -0.133 | 1, 32.39 | 0.018 | 0.895 |
| Body mass | 0.019 | 0.013 | 1.405 | 1, 108.91 | 1.813 | 0.181 |
| Sex (male) | 0.027 | 0.034 | 0.806 | 1, 129.07 | 0.623 | 0.432 |
| Cross-foster (yes) | 0.064 | 0.031 | 2.052 | 1, 110.91 | 4.114 | **0.045** |
| Hormone × Heating | -0.104 | 0.066 | -1.565 | 1, 125.20 | 2.367 | 0.127 |
| Hormone × Sex | 0.056 | 0.067 | 0.837 | 1, 127.42 | 0.669 | 0.415 |
| Heating × Sex | -0.027 | 0.068 | -0.409 | 1, 126.67 | 0.158 | 0.692 |
| Sample sizes: Day 8: n = 52 CO-non-heated, 39 CO-heated, 45 TH-non-heated, 37 TH-heated  Day 13: n = 47 CO-non-heated, 34 CO-heated, 37 TH-non-heated, 31 TH-heated | | | | | | |

**Table S7**. General linear mixed model (GLMM) on the effects of prenatal thyroid hormone elevation (TH/Control) and postnatal temperature elevation (heated/non-heated) on blood lipid peroxidation (malonaldehyde, MDA concentration) at (a) d8 and (b) d13 after hatching. Body mass and cross-fostering status (cross-fostered or not) have been included as covariates, and nests of origin and rearing as random intercepts. Sex was determined at d13. Main effects were reported from a model with no interactions. The significance tests were conducted using Kenward-Roger approximation on the degrees of freedom.

| (a) Day-8 MDA (n=172) | | | | | | |
| --- | --- | --- | --- | --- | --- | --- |
| Random effects: |  | **Variance** | **Std. Dev.** |  |  |  |
| Nest of origin (n=45) | Intercept | 0.003 | 0.056 |  |  |  |
| Nest of rearing (n=44) | Intercept | 0.017 | 0.130 |  |  |  |
| Residual |  | 0.043 | 0.208 |  |  |  |
| Fixed factors | **Estimate** | **SE** | **t** | **df** | **F** | **p** |
| Intercept | -2.607 | 0.182 | -14.347 |  |  |  |
| Hormone (TH) | -0.011 | 0.039 | -0.293 | 1, 26.37 | 0.083 | 0.775 |
| Heating (Non-heated) | -0.005 | 0.052 | -0.102 | 1, 35.09 | 0.010 | 0.920 |
| Body mass | -0.039 | 0.014 | -2.721 | 1, 149.63 | 6.992 | **0.009** |
| Cross-foster (yes) | -0.062 | 0.033 | -1.888 | 1, 123.13 | 3.523 | 0.063 |
| Hormone × Heating | -0.044 | 0.071 | -0.624 | 1, 144.79 | 0.380 | 0.539 |
| (b) Day-13 MDA (n=149) | | | | | | |
| Random effects: |  | **Variance** | **Std. Dev.** |  |  |  |
| Nest of origin (n=45) | Intercept | 0.008 | 0.087 |  |  |  |
| Nest of rearing (n=39) | Intercept | 0.024 | 0.156 |  |  |  |
| Residual |  | 0.016 | 0.126 |  |  |  |
| Fixed factors | **Estimate** | **SE** | **t** | **df** | **F** | **p** |
| Intercept | -3.094 | 0.171 | -18.095 |  |  |  |
| Hormone (TH) | -0.028 | 0.038 | -0.746 | 1, 26.28 | 0.544 | 0.467 |
| Heating (Non-heated) | -0.022 | 0.056 | -0.388 | 1, 31.19 | 0.150 | 0.702 |
| Body mass | <0.001 | 0.012 | -0.008 | 1, 141. 74 | <0.001 | 0.994 |
| Sex (male) | 0.005 | 0.027 | 0.188 | 1, 118.00 | 0.034 | 0.853 |
| Cross-foster (yes) | 0.033 | 0.023 | 1.436 | 1, 99.72 | 2.031 | 0.157 |
| Hormone × Heating | -0.094 | 0.052 | -1.817 | 1, 115.39 | 3.209 | 0.076 |
| Hormone × Sex | 0.020 | 0.052 | 0.387 | 1, 112.53 | 0.146 | 0.703 |
| Heating × Sex | -0.054 | 0.054 | -1.005 | 1, 120.32 | 0.976 | 0.325 |
| Sample sizes: Day 8: n = 52 CO-non-heated, 39 CO-heated, 45 TH-non-heated, 36 TH-heated  Day 13: n = 47 CO-non-heated, 34 CO-heated, 37 TH-non-heated, 31 TH-heated | | | | | | |


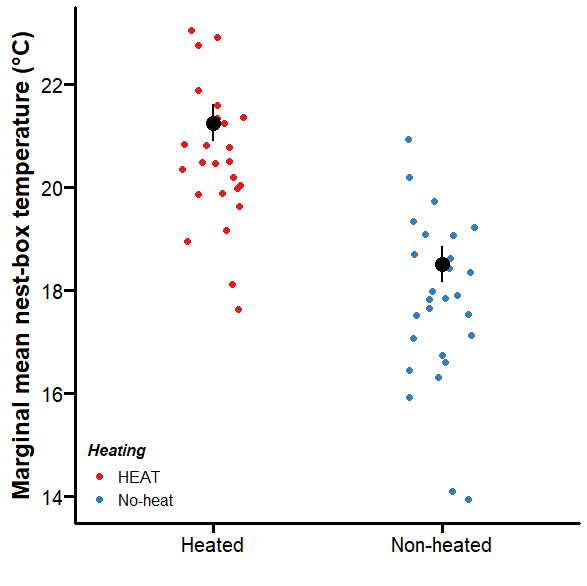


**Figure S1. Average nest-box temperatures across the period of heating treatment**. Small dots represent the average nest-box temperature from day 2 to day 8. Large black dots (mean±SE) represent marginal mean temperature controlled for date and iButton position.


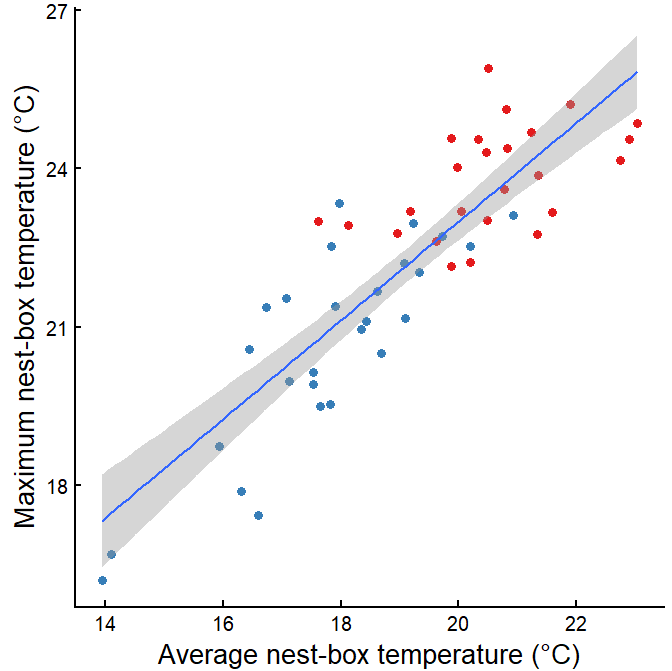

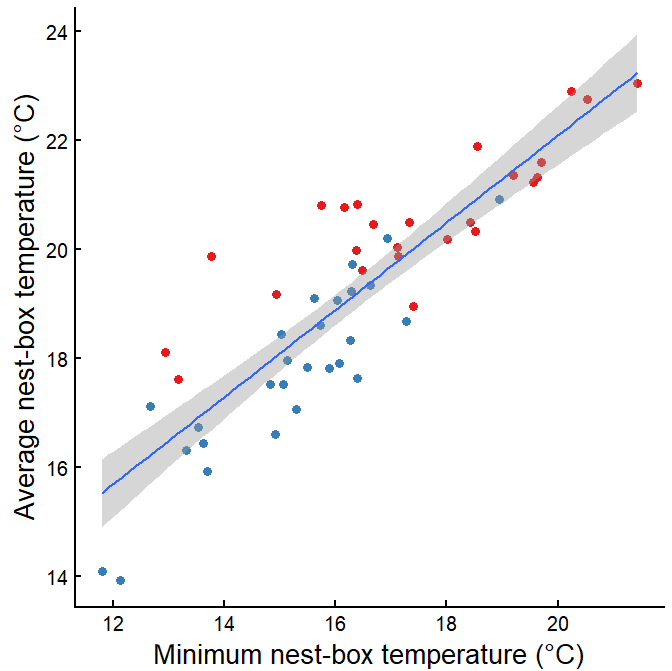

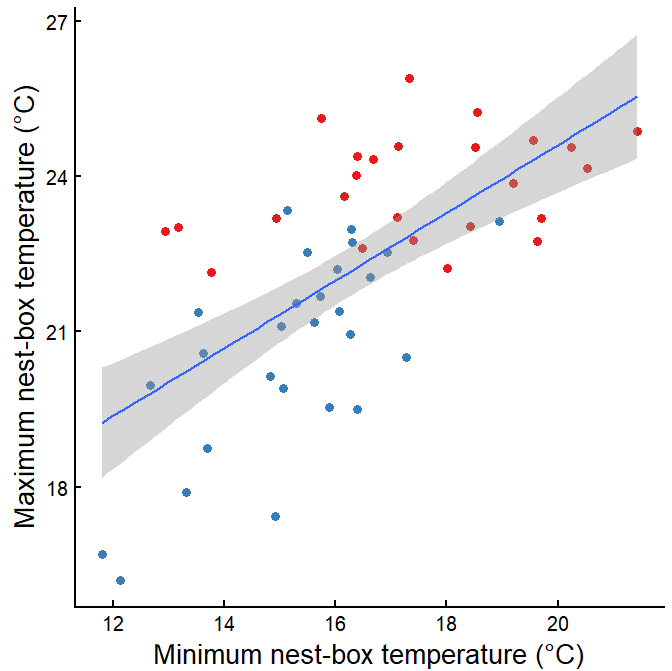


| Pearson’s r | Average | Maximum |
| --- | --- | --- |
| Minimum | **0.876** | **0.657** |
| Average |  | **0.858** |

**Figure S2. Correlations between minimum, average, and maximum nest-box temperature across the period of heating treatment. Red** and blue dots represent the heated and non-heated boxes, respectively.

**
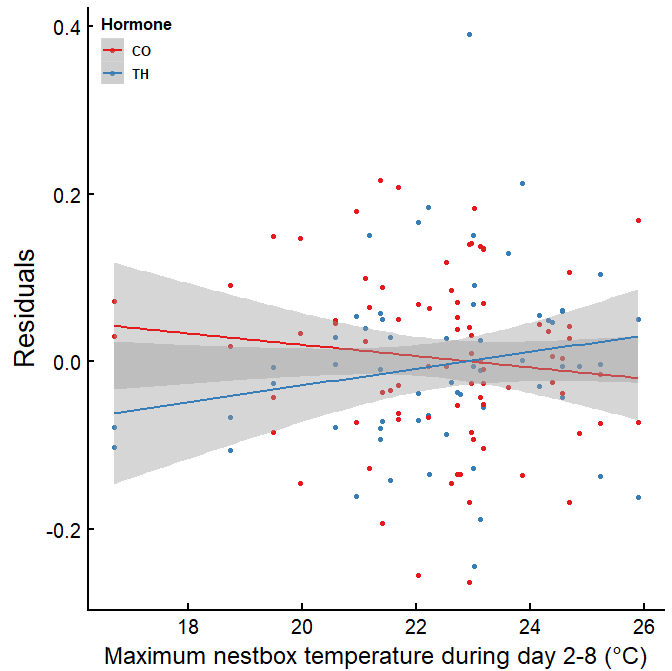

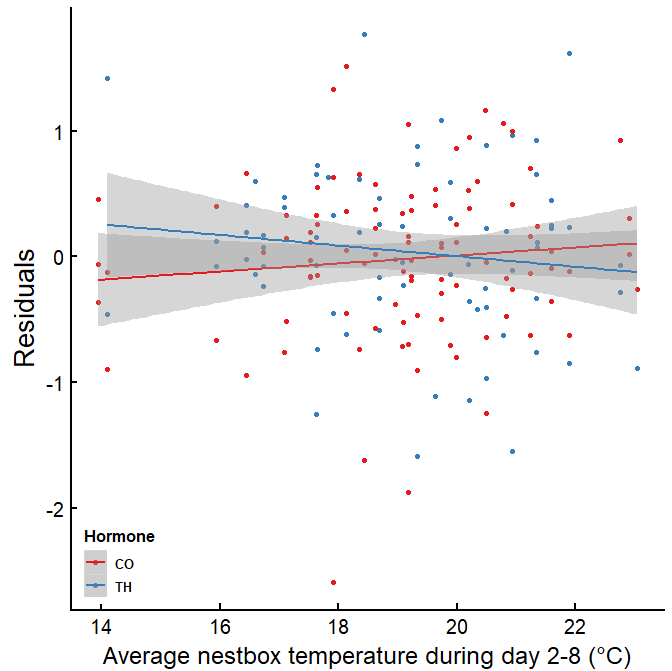
**

**Figure S3. Scatterplots of model residuals on nestling body mass (left panel) and blood malondialdehyde (MDA) concentrations across average (left panel) and maximum (right panel) nest-box temperature during the period of heating treatment (day 2 to day 8 after hatching).**

**Reference**

**Aljanabi SM, Martinez I.** 1997. Universal and rapid salt extraction of high quality genomic DNA for PCR-based techniques. Nucleic Acid Research **25:**4692-4693.

**Change H-W, Cheng C-A, Gu D-L, Chang C-C, Su S-H, Wen C-H, Chou Y-C, Chou T-C, Yao C-T, Tsai C-L, Cheng C-C.** 2008. High-throughput avian molecular sexing by SYBR green-based real-time PCR combined with melting curve analysis. BMC Biotechnology **8:**12.

**Ellegren H, Fridolsoon AK.** 1997. Male-driven evolution of DNA sequences in birds. Nature genetics **17:**182-184.

**Larsen S, Nielsen J, Hansen CN, Nielsen LB, Wibrand F, Stride N, Schroder HD, Boushel R, Helge JW, Dela F, Hey-Mogensen M.** 2012. Biomarkers of mitochondrial content in skeletal muscle healthy young human subjects. The Journal of Physiology **590:**3349-3360.

**Stier A, Bize P, Hsu B-Y, Ruuskanen S.** 2019. Plastic but repeatable: rapid adjustments of mitochondrial function and density during reproduction in a wild bird species. Biology Letters **15:**5.
